# Exome Sequencing Identifies Potentially Druggable Mutations in Nasopharyngeal Carcinoma

**DOI:** 10.1038/srep42980

**Published:** 2017-03-03

**Authors:** Yock Ping Chow, Lu Ping Tan, San Jiun Chai, Norazlin Abdul Aziz, Siew Woh Choo, Paul Vey Hong Lim, Rajadurai Pathmanathan, Noor Kaslina Mohd Kornain, Chee Lun Lum, Kin Choo Pua, Yoke Yeow Yap, Tee Yong Tan, Soo Hwang Teo, Alan Soo-Beng Khoo, Vyomesh Patel

**Affiliations:** 1 Cancer Research Malaysia, 47500 Subang Jaya, Selangor, Malaysia; 2 Molecular Pathology Unit, Cancer Research Centre, Institute for Medical Research, 50588 Kuala Lumpur, Malaysia; 3 Department of Oral and Craniofacial Sciences, Faculty of Dentistry, University of Malaya, 50603 Kuala Lumpur, Malaysia; 4 Tung Shin Hospital, 55100 Kuala Lumpur, Malaysia; 5 Sime Darby Medical Centre, 47500 Selangor, Malaysia; 6 Department of Pathology, Faculty of Medicine, University Teknologi MARA (UiTM), 47000 Sungai Buloh, Selangor, Malaysia; 7 Department of Otorhinolaryngology, Queen Elizabeth Hospital, 88200 Kota Kinabalu, Sabah, Malaysia; 8 Department of Otorhinolaryngology, Hospital Pulau Pinang, 10990 Penang, Malaysia; 9 Department of Surgery, Clinical Campus Faculty of Medicine and Health Sciences, University Putra Malaysia, Hospital Kuala Lumpur, 50586 Kuala Lumpur, Malaysia; 10 Department of Otorhinolaryngology, Head and Neck Surgery, Sarawak General Hospital, 53586 Kuching, Sarawak, Malaysia

## Abstract

In this study, we first performed whole exome sequencing of DNA from 10 untreated and clinically annotated fresh frozen nasopharyngeal carcinoma (NPC) biopsies and matched bloods to identify somatically mutated genes that may be amenable to targeted therapeutic strategies. We identified a total of 323 mutations which were either non-synonymous (n = 238) or synonymous (n = 85). Furthermore, our analysis revealed genes in key cancer pathways (DNA repair, cell cycle regulation, apoptosis, immune response, lipid signaling) were mutated, of which those in the lipid-signaling pathway were the most enriched. We next extended our analysis on a prioritized sub-set of 37 mutated genes plus top 5 mutated cancer genes listed in COSMIC using a custom designed HaloPlex target enrichment panel with an additional 88 NPC samples. Our analysis identified 160 additional non-synonymous mutations in 37/42 genes in 66/88 samples. Of these, 99/160 mutations within potentially druggable pathways were further selected for validation. Sanger sequencing revealed that 77/99 variants were true positives, giving an accuracy of 78%. Taken together, our study indicated that ~72% (n = 71/98) of NPC samples harbored mutations in one of the four cancer pathways (EGFR-PI3K-Akt-mTOR, NOTCH, NF-κB, DNA repair) which may be potentially useful as predictive biomarkers of response to matched targeted therapies.

Targeted therapies with higher selectivity and minimal toxicity are now frequently used as standard-of-care for treating several human cancers that include lung^1^, breast^2^ and colorectal^3^ harboring specific genetic alterations. By contrast, treatment for nasopharyngeal carcinoma (NPC) patients still remains limited to combination of radiotherapy and cytotoxic agents for example, cisplatin, 5-FU, palictaxel and gemcitabine^4^. To this end, several groups have also explored the potential of using anti-EGFR monoclonal antibodies (cetuximab and nimotuzumab) with radiotherapy and chemotherapy in improving outcome of NPC patients but the results thus far, have been mixed^5^,^6^,^7^. It is worth mentioning that newly diagnosed NPC patients generally respond favorably to available first line treatment, but many face a risk of developing debilitating side effects such as mucositis that severely impacts the quality of life^8^, although these are reduced by improved precision of radiotherapy^9^. Even with the best treatment, up to 30% of cases end up with treatment failure, with distal control being the major challenge^9^.

Therefore, there is a pressing unmet need to expand the current treatment options, particularly for metastatic disease, preferably through biomarkers guided approach for NPC patients to improve clinical outcome and overall survival. This is especially important in Malaysia where this cancer remains the fifth most common^10^, often diagnosed at advanced stages III and IV^11^ and with the highest incidence rates amongst the local indigenous population compared to neighbouring countries^12^.

While deregulated expression of oncogenes and tumor suppressor genes are now known to contribute to cancer, they do not always offer sufficient information for guiding treatment decisions. For example, lung cancer patients expressing high epidermal growth factor receptor (EGFR) levels may not all benefit from anti-EGFR therapies (i.e. erlotinib/gefitinib), while those harboring gain of function mutations which led to constitutive activation of this mitogenic pathway are likely to be responsive and confer prolonged median survival^13^,^14^. In this regard, *EGFR*-mutant advanced lung cancer patients are reported to have a higher 5-year survival (14.6%)^13^ with anti-EGFR therapies when compared to unselected patients (8.4%)^14^. However, the mutational status of a single gene alone can in some cases, fail to provide benefit with this class of targeted therapy. For instance, *EGFR*-mutant colorectal and lung cancer patients are essentially resistant to anti-EGFR therapies if accompanied by *KRAS* mutation^15^,^16^. Notwithstanding, the successful translation of mutation status in guiding oncology treatment decision has gained increasing attention and the growth in this field has been accelerated with the advent of next generation sequencing^17^,^18^.

It follows that with the current next generation sequencing capabilities, we are now able to exquisitely tease out the complex genetic information underlying cancer development and concurrently identify relevant gene signatures that are unique to each tumor and link these to drug response with the clear view of identifying new therapies as treatment options^19^,^20^,^21^. Exome sequencing is a cost-effective approach to identify mutational profile, and has been used extensively for discovering driver mutations as well as to uncover new potential treatment strategies for various cancer types. For instance, gallbladder carcinoma which has limited treatment options and poor survival, now may be amenable to tyrosine kinase inhibitors, in which genes involved in the ERBB pathway were found recurrently mutated (n = 21/57; 36.8%) by employing exome sequencing^22^. Given that genome wide sequencing data are meaningful to identify genomic similarities between different cancers that may be sensitive to the same treatment regimen, numerous high throughput cancer sequencing projects have been conducted, especially by The Cancer Genome Atlas network on various cancer types, including breast^23^, acute myeloid leukemia^24^, lung^25^, head and neck squamous cell carcinomas^26^ and many others. Indeed, several ongoing clinical trials for example, NCI Molecular Analysis for Therapy Choice (MATCH) and FOCUS4, now employ predetermined mutational profile of the cancer before giving the relevant targeted therapy^27^.

To date, limited information is available on key molecules and actionable targets that can be exploited for predicting drug sensitivity to existing therapeutics and/or developing new therapies for NPC. In this regard, earlier studies reported that hotspot mutations across 19 common oncogenes/tumor suppressor genes (e.g. *TP53, KIT, KRAS*) are infrequent in NPC tumors^28^, highlighting that genome wide screening remains pivotal for enhancing our current understanding on aberrant mutational events that likely drives NPC pathogenesis. Recently, Lin *et al*.^29^ has identified several potential targets for NPC based on genomic alterations using the exome sequencing approach, that may form the basis for exploring targeted therapies. Therefore, to make further inroads into identifying genetic changes underpinning NPC pathogenesis and consequently druggable targets, we performed whole exome sequencing on a small cohort (n = 10) of untreated freshly frozen and clinically annotated NPC tumor biopsies and from the emerging data, we screened 42 prioritized candidate genes in additional 88 NPC tumor biopsies collected from local hospitals, to determine their frequency of occurrence in multi-ethnic Malaysian NPC patients. Our data demonstrated that several genes in key cancer pathways (EGFR-PI3K-Akt-mTOR, NOTCH, NF-κB, DNA repair) were mutated, suggesting their potential use as predictive markers for targeted therapies to improve survival and outcome of NPC patients.

## Results

### Patient demographics

Clinicopathological details of the NPC patient samples are summarized in Table 1 and Supplementary Table 1. From the 98 tumors that were included in the Discovery (n = 10) and Prevalence cohorts (n = 88), the majority were from patients of Chinese origin (70%), while the remaining ~30% were Malay, Indigenous and other sub-category. Notably, a higher ratio of males to females (3.7:1) was embedded in our sample demographics and consistent with the ~3-folds higher incidence of NPC in males than females^30^. The median age at diagnosis was 51 years, while a notable number (7%) were below 35 years of age upon diagnosis.

**Figure 1:**
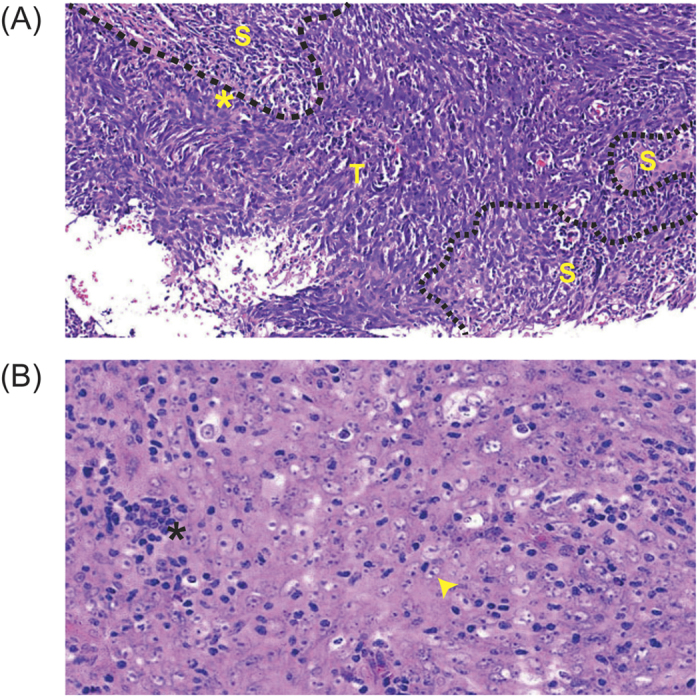
Representative NPC cases used in this study. **(A**) Undifferentiated and non-keratinizing carcinoma of nasopharynx (WHO Type II). The tumor cells are elongated and spindle shaped, exhibit dark nuclei and invade the stroma forming syncytial sheets (*). A stromal infiltrate of lymphocytes is apparent and the broken line represents boundary between stroma (S) and tumor (T) (20X). (**B**) Undifferentiated carcinoma of nasopharynx (WHO Type III). The tumor cells are oval and rounded, with large vesicular nuclei and prominent nucleoli (arrow head). The growth pattern is largely syncytial and occasional small lymphocytes (*) are observed intermingled with tumour cells (40X).

NPC is a malignancy of the epithelium that shows a variable degree of squamous differentiation where the vast majority of tumors, are undifferentiated without evidence of keratinization and are typically WHO types II and III. Furthermore, the association with *Epstein-Barr* Virus (EBV) is consistent across all types of NPC although the viral presence may be difficult to demonstrate in those lesions that are WHO type I^31^. The majority of the samples in our study cohort were categorized as WHO Type III NPC (83%) and diagnosed at an advanced stages of the disease (III and IV), in accordance with earlier reports^31^,^32^. Detailed histopathological evaluation of all tissues revealed that the tumor content was approximately 40–100% and this is detailed together with the clinical TNM classification in Supplementary Table 1. Figure 1A shows a representative case of a Type II, undifferentiated and non-keratinizing carcinoma of the nasopharynx used in this study where the presence of elongated and spindle shaped tumor cells exhibiting dark nuclei are seen invading the stroma, forming syncytial sheets in intimate relationship with stromal infiltrates of lymphocytes. Figure 1B depicts an example of an undifferentiated Type III carcinoma of the nasopharynx used in this study where the tumor cells are observed to be oval and rounded, with eosinphilic cytoplasm and large vesicular nuclei and prominent nucleoli. The growth pattern is largely syncytial and occasional small lymphocytes are observed intermingled with tumor cells. Of note, as a large proportion of patients failed to return for follow up, either after the initial diagnosis or treatment, the prognostic value of the candidate mutations could not be assessed.

### Identification of somatic mutation by whole exome sequencing

**Figure 2:**
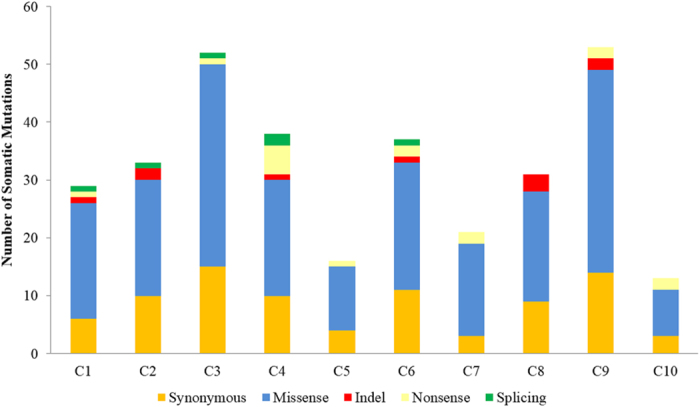
Distribution of somatic mutations identified from the Discovery set. Whole exome sequencing of the samples (C1-C10), a total of 323 somatic variants affecting 308 genes were identified, of which 238 were non-synonymous (10 indel, 16 nonsense, 6 spicing, 206 missense), and 85 were synonymous.

**Figure 3:**
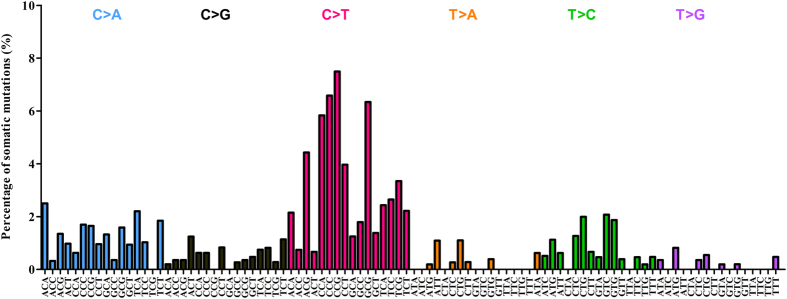
Distribution of nucleotide changes of the somatic mutations identified from the Discovery set. Mutations identified are shown in their trinucleotide context, which is defined by the substituted base flanked by immediate 5′ and 3′ bases. Analysis based on 96-substitution classification revealed that C to T transition is shown to be predominant in the NPC Discovery set as well as an enrichment in NpCpGp context, a signature which is associated with spontaneous deamination by APOBEC3B.

**Figure 4:**
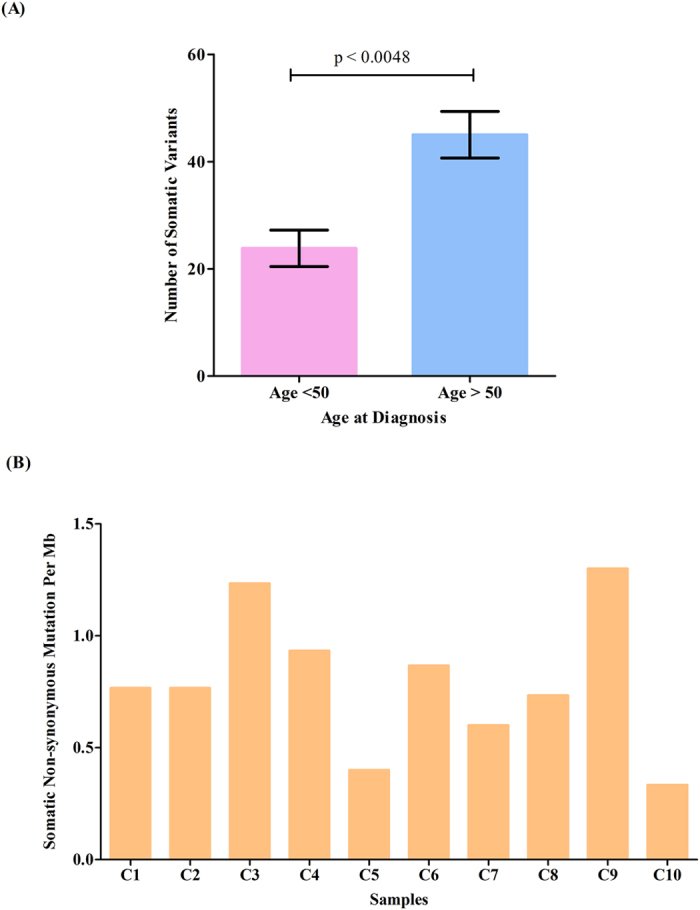
Association of somatic mutations with patients’ age and the mutation rate in NPC. (**A**) NPC patients aged >50 years are shown to have a significantly higher number of variants as compared to those <50 years (*p* = 0.0048, unpaired T test). (**B)** A nonsynonymous somatic variant rate of 0.79 per Mb (range 0.33–1.3) was identified in the Discovery set.

To explore the mutational landscape of NPC, we first performed whole exome sequencing on DNA extracted from 10 untreated NPC biopsies with at least 80% tumor content (Supplementary Table 1) and matched normal blood. The mean depth coverage was 110X for the tumors and 109X for matching normal (blood) (Supplementary Table 2). All samples had >96% of targeted bases represented by at least 10 reads (Supplementary Table 2). After filtering the exome data to exclude germline variants and reported polymorphisms, a total of 323 somatic mutations (206 missense, 85 synonymous, 16 nonsense, 6 splicing, 10 indel) affecting 308 genes were identified from the protein coding regions (Supplementary Table 3). A total of 30 candidate mutated genes fulfilling one of the following criteria: (1) cancer gene census; (2) mutations affecting functional domains; (3) predicted deleterious by SIFT or Polyphen2; (4) reported to be involved in tumorigenesis; were selected for validation by Sanger sequencing. Sequence validation revealed that 29/30 variants were confirmed true positive as somatic mutations (present in tumor and absent in matched normal DNA), yielding a true positive rate of ~97% (Supplementary Table 4). A mean of 32 somatic variants per patient (range 13–53 variants) were detected in our Discovery set, with the majority being identified as synonymous and missense (Fig. 2). Mutation spectra analysis with regards to the trinucleotide context showed that the C:G > T:A transitions was the most predominant (n = 164/313) in our Discovery set of 10 NPC tumors. Notably, ~50% of the trinucleotide alterations were occurring at CpG dinucleotides (Fig. 3), a signature that has now been reported to be associated with spontaneous DNA cytidine deaminase activity of APOBEC3B^33^,^34^, and consistent with observations recently reported^29^. When the age of the patients was factored into our data, those over 50 years of age had significantly more variants (range 37–53) as compared to those below aged 50 (range 13–33) (Fig. 4A) (*p*-value = 0.0048), which likely support the notion that mutational accumulation increases with age and can add value as a predictor of a poorer outcome^35^,^36^. Furthermore, we did not observe notable correlation between the number of somatic mutations and tumor stages (early stage II compared to late stages III & IV, *p*-value* = *0.26). An average nonsynonymous somatic mutation rate of 0.79 per Mb (range 0.33–1.3) was found in our Discovery set (Fig. 4B). Although recurring mutation (same mutation in multiple samples) was not observed in our analysis of 10 NPC exomes, 6 genes were found to be commonly mutated in multiple samples, i.e. *CXorf22* (p.H235Y, p.A940T), *MYH1* (p.A338T, p.R109C), *N4BP1* (p.G685E, p.R597X), *WDR87* (p.D2021E, p.M1261I), *TRAF3* (p.R163X, p.S9fs), and *KCNN3* (p.P81delinsQQQQQP, p.L66H).

### Enriched altered pathways in NPC

**Figure 5:**
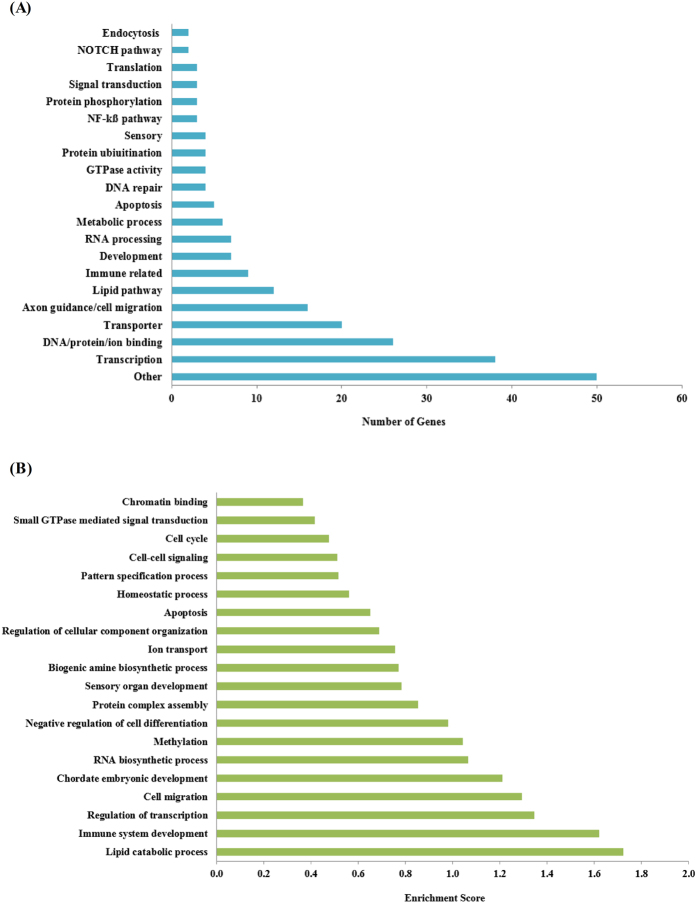
Over-represented biological processes and pathways associated with mutated genes in NPC. (**A**) Mutated genes giving rise to altered protein expression identified and those encoding transcription factors where the most commonly impacted in the Discovery set. (**B**) Functional clustering using DAVID revealed that lipid catabolic process was the most significantly enriched pathway identified from protein altering mutated genes in the Discovery set.

As depicted in Fig. 5A, classification of genes with protein-altering mutations using gene ontology biological process terms showed that transcription factors were the most significant enriched class of mutated genes (38/238 genes; 16%), in which all the samples analyzed (Discovery set only) harbored at least one mutated transcription factor. Among those, *MED12, CDX2, MLL* and *MLL3* are cancer census genes, which are found frequently mutated in other cancers (COSMIC). For example, *MLL3* ranked the six most commonly mutated gene in breast cancer^23^ (7%), but have not been reported for NPC. Other notable features included signaling pathways associated with the several of the mutated genes, for example, DNA repair (*ATM, TP53, FEN1*), NF-κβ pathway (*TRAF3, NLRP6, CC2D1A*), NOTCH (*NOTCH2, DLL1, FBXW7*) and lipid signaling (*ASPG, CHKB, CPS1, IMPAD1, LPIN3, OXCT1, PHLDB2, PLCG1, PLCH1, PLCH2, PRKCZ, SYNJ1*), many of which may have potential therapeutic implications.

Based on DAVID functional clustering analysis, the genes with somatic variants in our study were most enriched in the lipid catabolic process (enrichment score = 1.72), followed by immune system development (enrichment score = 1.62) and regulation of transcription (enrichment score = 1.35) (Fig. 5B). Overall, our analysis revealed that 7/10 samples of the Discovery set harbored mutations implicated in lipid signaling, NOTCH, NF-κB pathway and DNA repair mechanisms. As these pathways are druggable and that the predictive value of these somatic mutations can potentially identify NPC patients likely to benefit from selected targeted therapy, further investigation is warranted.

### Haloplex targeted resequencing

To identify frequently mutated genes implicated in NPC, a total of 37 candidate genes (those known to be implicated in cancer pathogenesis) and 5 mutated cancer genes listed in COSMIC (*EGFR, CDKN2A, PTEN, KRAS, PIK3CA*), were prioritized for further assessment with an additional 88 NPC cases. Targeted deep sequencing approach was carried out with a mean read depth of 2183X for the targeted regions and an average of 95.8% of the analyzable targeted bases being represented by at least 100 reads (Supplementary Table 6).

Overall, a total of 9987 variants were detected in our Prevalence set (Supplementary Table 7) and in order to evaluate the validity of recurring variants (same mutation occurring in multiple samples), 12 of the recurring variants from 25 NPC cases leading to 28 PCR reactions were randomly selected for evaluation by Sanger sequencing (Supplementary Table 8). Comparing recurring variants detected in 3 or less samples (≤3) to those detected in more than 3 samples (>3), we observed that the true positive rate differed from 100% to 24% (11/11 to 4/17, Supplementary Table 8). As a consequent of this observation, recurring variants detected in >3 samples were omitted from downstream analysis to eliminate false positive variants, which likely arises due to PCR artefacts. After removal of synonymous variants and known polymorphisms, except for those reported in the COSMIC database, our analysis detected a total of 160 putative non-synonymous variants affecting 37/42 genes in 66/88 NPC patients (Supplementary Table 9).

Of these 160 non-synonymous variants, those in the top five most frequently mutated genes, namely *TP53, NOTCH2, PLXNB3, ATM*, and *PPP1R15A*, all recurring variants, and together with some randomly selected variants were prioritized for validation by Sanger sequencing. Samples with no remaining DNA or PCR products failing to be amplified were omitted from validation by Sanger sequencing. Sanger sequencing validated variants with unavailable germline DNA to determine somatic origin were denoted as somatic/germline. Overall, Sanger sequencing results confirmed 78% (77/99) putative non-synonymous variants identified by haloplex as true positives (Supplementary Table 9).

For the selected top five most frequently mutated genes detected in our prevalence set, *TP53* was confirmed with 9 somatic and 1 somatic/germline variants. *NOTCH2* was confirmed with 2 somatic, 7 germline and 1 somatic/germline variants. For *PLXNB3* variants, 3 were somatic, 1 was germline and 1 was somatic/germline. *ATM* was confirmed with 7 germline variants while *PPP1R15A* confirmed with 1 germline variant (Supplementary Table 9). Among the 24 recurring variants, only 16 candidates were validated by Sanger sequencing in all samples harbouring the putative variants. Sanger sequencing confirmed 2/16 were recurring somatic variants, 6/16 were recurring germline derivatives, 2/16 with mixed profile, and remaining 6 were noted as false (Supplementary Table 9).

**Figure 6:**
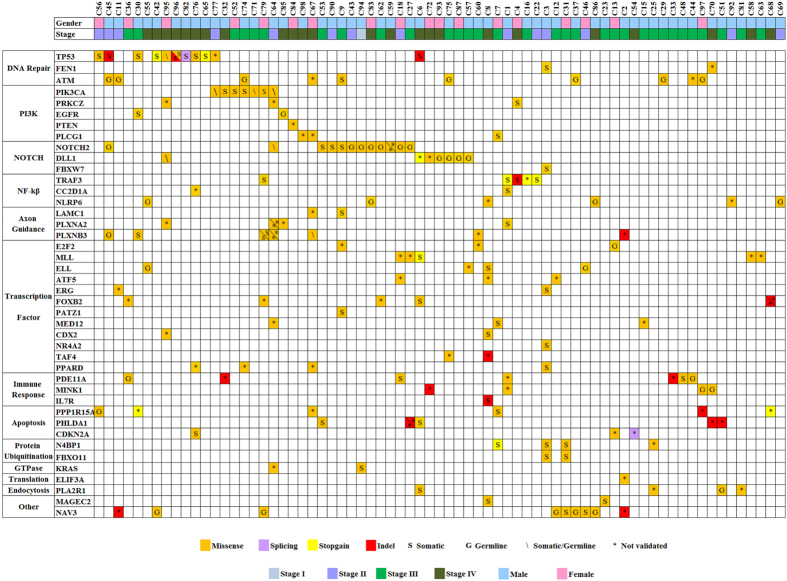
Mutational landscape of candidate genes detected in the NPC tumor cohort. Each column represents an individual affected case, and each row denotes a specific gene assigned to one of the labeled functional categories (left panel). Each type of mutation class is indicated by color, symbol G indicated validated germline variant, symbol S indicated validated somatic variant, symbol \ indicated somatic/germline variant, and symbol * indicated non-validated variant. Patients’ gender and clinical staging are shown in the top panel.

In summary, our findings fall broadly in concordance with those of Lin *et al*.^29^ reporting that NPC genomic landscape harbors a very limited number of somatic variants as compared to other types of human cancers. Nonetheless, from our combined Discovery and Prevalence sets, 72% (71/98) of NPC cases had frequently mutated genes involved in cancer pathways (Fig. 6). The most affected pathways include the DNA repair (i.e. *TP53, ATM, FEN1*, n = 22/98, ~22%), NOTCH (i.e. *NOTCH2, DLL1, FBXW7*, n = 20/98, ~20%), EGFR/PI3K/Akt/mTOR (i.e. *PIK3CA, PRKCZ, EGFR, PTEN, PLCG1*, n = 15/98, ~15%) and the NF-κB pathway (i.e. *TRAF3, CC2D1A, NLRP6*, n = 12/98, ~12%) (Fig. 6). Variants found in these pathways, irrespective of germline or somatic, may potentially serve as predictive biomarkers for targeted cancer therapies. Of interest, although we found no significant differences in the number of non-synonymous variants among different disease stage (data not shown), we did note that 44% (4/9) of Stage 4 C metastatic NPC cases had mutations in the DNA damage response compared to 20% (18/89) in non-metastatic cases (Supplementary Figure 1). Taken together, our findings revealed that a subset of NPC patients may be amenable to biomarker-guided therapies targeting EGFR-PI3K-Akt-mTOR, NOTCH, NF-κB signaling and DNA repair pathways.

## Discussion

It is well accepted that cancer arises from somatically acquired mutations and the identity of these affected molecules can lead to developing actionable targeted therapies. Indeed, several somatic mutations have now been developed into molecular screening tools (companion tests) for guiding precision therapies^13^,^14^,^37^,^38^. For example, the current practice of matching non-small cell lung cancer patients carrying *EGFR* mutations to afatinib/osimertinib/erlotinib small molecule class of therapies, whereas those with wildtype alleles to be administered with conventional chemotherapies^13^,^14^,^38^. This also includes matching those lung cancer patients who may have acquired the *EGFR* T790M mutation conferring resistance while on prior therapy with EGFR small molecule inhibitors and prescribing new generation of targeted therapies to help override this. For example, AZD9291, a selective oral EGFR tyrosine kinase inhibitor was found to be active in patients with *EGFR* T790M mutation^39^. Furthermore, the presence of the *BRAF* V600 variant in melanoma patients predicts benefit with FDA approved small molecule therapies, and vermurafenib has been shown to give benefit to *BRAF* V600E positive metastatic melanoma patients^37^,^40^. Unlike other solid tumors that have well characterized genomic profiles via next generation sequencing (e.g. breast and lung cancer)^23^,^25^, little is known about the genetic landscape underpinning NPC pathogenesis. This likely reflects the fact that to date, limited options for targeted therapies for NPC and consequently, there is considerable interest in identifying variants that can be predictive of treatment response, allowing NPC patients to be better matched with anti-cancer therapies based on the tumor genotype. In this study, genes which are involved in several druggable pathways, namely the EGFR-PI3K-Akt-mTOR, NOTCH, NF-κB signaling and DNA repair pathways were found to be commonly mutated in a large multi-ethnic Malaysian NPC samples cohort (n = 98).

Defects in DNA repair mechanism represents key events in tumorigenesis, and is most commonly observed in our samples set (n = 22/98) especially in metastatic NPC (n = 4/9). *TP53*, referred to as the guardian of the genome, essentially functions as a tumor suppressor by maintaining genome stability and integrity through cell cycle checkpoints^41^. While loss of function mutations for this gene are the most frequently reported for human cancers (COSMIC; http://www.cancer.sanger.ac.uk), for NPC, several studies have reported a lower frequency, broadly in the range of ~10% using both the Sanger^42^,^43^,^44^,^45^,^46^ and exome sequencing approaches^29^. In concordance with these earlier studies, we noted that in our study *TP53* was mutated at a frequency of ~11% (n = 11/98). Despite a lower prevalence of *TP53* mutations in our NPC samples as compared to other tumor types, loss of function in *TP53* nonetheless represents a key genetic defect underpinning NPC pathogenesis. In total, we detected 12 mutations in 11 patients, which include 5 truncating mutations (p. R342fs, p.R213X, p.R306X, p.L330fs, p.S95fs), 1 splicing mutation (c.375 + 1 G > C), and 6 missense mutations (p.R248Q, p.R282W, p.G245D, p.Y126H, p.V31I, p.R273C). Of note, the *TP53* variant p.S95fs, was initially detected as p.S95Y in our Haloplex analysis, however validation by Sanger sequencing identified this as a frameshift mutation, suggesting confirmation with this second method should be incorporated into clinical diagnosis for guiding treatment decision. Interestingly, *TP53* has been reported to be involved in metastasis by inducing cell invasion and migration processes^47^, and *TP53* deficient tumors are associated with a poorer outcome^48^,^49^,^50^,^51^. The prognostic value of *TP53* mutations in NPC is yet to be determined and likely an area of investigation that has value. From a treatment perspective, nutlin-3a, a small-molecule that inhibits TP53-MDM2 interaction, can reactivate TP53 function^52^, thereby inducing cell cycle arrest and apoptosis^53^. Given that nearly 90% of NPC harbour wildtype *TP53*, nutlin-3a has potential as a therapeutic agent for NPC patients^54^. Preclinical studies have shown that nutlin-3a is broadly effective against cancer cells expressing TP53 either as a single agent^55^,^56^, or in combination with chemotherapeutic agent, such as cisplatin in NPC, lung and ovarian cancer^54^,^55^,^57^. In addition to somatic *TP53* mutations, our data revealed non-synonymous mutations in *ATM*, another DNA repair gene in a subset of NPC (n = 10/98, ~10%). Although the pathogenic roles of these mutations currently remain unknown, PARP inhibitors have recently been reported to give benefit to patients harboring germline defects in key DNA repairs genes, including *BRCA, ATM, CHEK2* and *TP53*^58^,^59^,^60^,^61^,^62^. Given that olaparib has recently been approved for the treatment of advanced ovarian cancer with deleterious *BRCA* mutations^58^,^63^, this may serve as a therapeutic window with FDA approved therapies for NPC patients with similar gene defects. The presence of mutations of the DNA damage pathway in NPC raises the possibility of using drugs targeting the pathway which could lead to synthetic lethality^64^, particularly for metastatic NPC where treatment options are limited.

From our analysis, we noted that *NOTCH2* was the most frequently mutated gene (n = 12/98; 12%). Also, a total of 20/98 samples (~20%) harbored mutation in genes associated with the NOTCH pathway (*FBXW7*: n = 1; *NOTCH2:* n = 12, *DLL1*: n = 7). This indicates that this pathway is the second most frequently mutated, and serve as promising treatment approach for NPC patients. While the association of NOTCH signaling and NPC pathogenesis remains unclear, our findings is the first to report *FBXW7* and *DLL1* mutations in this cancer type. Notwithstanding, *FBXW7* is a key component of the NOTCH pathway and functions by mediating ubiquitination and proteasomal degradation of several oncoproteins, including cyclin E, Notch, Jun and c-myc by forming ubiquitin ligase complex^65^,^66^. As loss of function mutations in *FBXW7* have been reported in several malignancies to be associated with resistance to various drugs for example, γ-secretase inhibitor MRK-003 in T-ALL^67^, palictaxel in breast cancer^68^ and trastuzumab in gastric cancer^69^, suggesting that the utility of *FBXW7* (p.S558F) to predict drug sensitivity in NPC warrants further investigation. While DLL1 functions as one of several ligands for NOTCH receptors, the pathogenic impact of mutations detected in our NPC samples remains unexplored. Given that *NOTCH2, FBXW7* and *DLL1* are key components in NOTCH signaling and have predictive value in cancer therapies, the effect of these mutations in NPC is yet to be elucidated.

Our mutational analysis also showed a trend towards an enrichment in the EGFR/PI3K/Akt/mTOR pathway where mutations in *PIK3CA* (n = 7), *PLCG1* (n = 3), *EGFR* (n = 2), *PTEN* (n = 1), and *PRKCZ* (n = 3) were identified in ~15% of our sample cohort. While *PIK3CA* mutations have now been reported to be present in 25–30% of human cancers including colorectal, gastric, oral squamous and brain^70^,^71^,^72^, this frequency is at a lower rate for NPC (5–9%)^29^,^73^,^74^, despite reported being amplified and overexpressed in ~70% of lesions analyzed^75^. We did not detect *PIK3CA* mutation in our discovery set, however 2 commonly found activating mutations in hotspot exon 9 (p.E542K: n = 2; p.E545K: n = 3) were noted in our validation cohort. To this end, gain of function mutations in *PIK3CA* can result in constitutive activation of the PI3K-Akt-mTOR signaling axis, which then promotes key hallmarks of oncogenesis, including proliferation, metastasis, angiogenesis and chemoresistance^76^,^77^,^78^. It follows that the presence of *PIK3CA* mutations is being exploited as a predictive marker of benefit as indicated in a recent clinical trial of PI3K/Akt/mTOR inhibitors on patients with advanced cancers, where a higher response rate was observed in patients with the mutation as compared to those without^79^,^80^,^81^. Also, everolimus for targeting tumors with *PIK3CA* mutations is currently under clinical evaluation (https://clinicaltrials.gov/). Taken together, the presence of *PIK3CA* mutations in our samples cohort suggests that a substantial number of NPC patients can likely benefit from therapies that target the PI3K/Akt/mTOR pathway. Beyond that, other mutations which fall into the EGFR-PI3K-Akt-mTOR pathway may also have therapeutic values. We detected 2 novel mutations in *EGFR* (p.L49F, p.K253R) and may provide the basis for testing current FDA approved anti-EGFR therapies. Also, both *PRKCZ* and *PLCG1* are intracellular receptor mediators for EGFR, PI3K, and VEGFR tyrosine activators^82^,^83^,^84^, which are involved in carcinogenesis by promoting cell growth, invasion and migration^85^,^86^,^87^. Hence, mutations detected in *PLCG1* (p.R187W, p.I1092V, p.R1229W) and *PRKCZ* (p.V283L, p.F517V, p.T99P), (encoding phospholipase Cγ1 and protein kinase C ζ, respectively), may hold value as predictive markers for anti-PI3K/Akt/mTOR therapies.

NF-κB represents an important transcription factor involved in the regulation of proliferation, apoptosis, metastasis and angiogenesis^88^,^89^. *TRAF3* is among the player negatively regulating canonical and non-canonical NF-κB activity and in mediating immune response by interacting with *TNFR* superfamily^90^. With regards to NPC, *TRAF3* truncating mutations have been reported in NPC C666 cells (c.415delA), as well as in 1/33 primary NPC tumors (p.N139MfsX20) suggesting a causal role in disease pathogenesis^91^. In addition, other NF-κB pathway associated genes, i.e. *A20* and *TRAF2* were also found mutated^91^. Beyond that, inactivation of *TRAF3* function may mediate NPC tumor cells survival upon *Epstein-Barr* virus infection by suppressing the production of interferon immune responses^91^. In this study, we detected 5 non-synonymous variants in *TRAF3* that included 3 nonsense (p.Q114X, p.R163X, p.R505X), 1 frameshift deletion (p.S9fs) and 1 missense (p.G480E) in 5/98 samples (~5%). On closer assessment of the sequence harboring these defects, we noted that the nonsense and deletion would have resulted in the loss of the TRAF3 domain, essential for negatively regulating NF-κβ may lead to increased activity of the pathway^92^,^93^,^94^,^95^. Several studies have now demonstrated that targeting the NF-κB pathway is a promising treatment strategy for a variety of cancers such as breast^96^, colorectal^97^, and head and neck^98^. Most notably, bortezomib, which inhibits NF-κB activity by blocking IκB degradation^99^, has been approved for the treatment of multiple myeloma^100^,^101^. Over-expression of NF-κB has been reported to be associated with poorer prognosis in NPC^102^,^103^ and therefore supporting the rational of exploiting NF-κB inhibitor as potential treatment strategy for NPC. Previous study has reported NF-κB inhibitors DHMEQ and BAY 11-7082, by blocking the nuclear translocation of activated NF-κB were effective in inhibiting NPC HK1 cell growth, induced apoptosis and abrogated their invasiveness and anchorage independent growth^104^. Furthermore, recent study by Peng *et al*.^105^ also showed that andrographolide inhibited proliferation and invasiveness of NPC HK1 cells via suppressing NF-κB transcriptional activity, thus providing a rational for the possibility of utilizing the NF-κB activation status to stratify NPC patients who may be more likely to benefit therapy with NF-κB inhibitors. From our observations, there is the possibility of incorporating *TRAF3* loss of function mutations as predictive biomarker of response to NF-κB inhibitors, and warrants further investigation.

Overall, alterations impacting EGFR-PI3K-Akt-mTOR, NOTCH, NF-κB signaling and DNA repair pathways accounted for ~72% (n = 71/98) of the NPC samples analyzed, and may be useful to predict clinical benefit in NPC patients. Among the potential matched drug-gene candidates including NF-κB inhibitors (*TRAF3*), NOTCH inhibitors (*NOTCH2, FBXW7*), nutlin-3a (*TP53*), PARP inhibitors (*TP53, ATM*), AKT-PI3K-mTOR inhibitors (*PIK3CA, PRKCZ, PLCG1*), and EGFR inhibitors (*EGFR*). Further investigation is warranted to validate these targets and to provide a more defined framework for clinical evaluation in NPC patients who may likely have exhausted the available standard of care therapies as well as newly diagnosed NPC patients. However, it is worth mentioning that validation of some of the observed mutations using cellular based or mouse xenograft models currently represents a major challenge as many of the available NPC cell lines are now reported to be contaminated with HeLa cells^106^,^107^.

## Materials and Methods

### Patient samples

This study was conducted in accordance with relevant guidelines and regulations by the ethics committee as indicated. Fresh frozen primary biopsies and matched normal blood samples from newly diagnosed and untreated NPC patients were collected from Tung Shin Hospital (TSH; Kuala Lumpur, Malaysia), Kuala Lumpur Hospital (KLH; Kuala Lumpur, Malaysia), Penang Hospital (PH; Penang, Malaysia), Queen Elizabeth Sabah Hospital (QESH; Sabah, Malaysia), and Sarawak General Hospital (SGH; Sarawak, Malaysia). Samples from TSH were obtained with approval from the ethics committee and signed informed consent from patients. Samples from KLH, PH, QESH and SGH were obtained with approval from the Medical Research and Ethics Committee, Ministry of Health Malaysia (NMRR-12-1203-14027) and signed informed consent from each patient.

### Sample preparation

For this study, a total of 10 cases were included in the initial Discovery set and 88 for the Prevalence set. Each sample after embedding in OCT were, cryosectioned using a cryostat (Leica Biosystems, IL, USA). For each sample, a 5 μm cryosection reference slide was made prior and after the collection of ~20 cryosections (each ~25 μm) in 1.5 mL microcentrifuge tubes. All reference slides were stained with hematoxylin and eosin (H&E) followed by histopathological evaluation by pathologists (RP and NKMK) to confirm the pathogenesis of the lesions including the levels of tumor cells presents following the classification described by Shanmugaratnam & Sobin (1978)^108^.Only those cryosections collected where the tumor content was confirmed to be ~40–70% were processed for DNA extraction. Clinicopathological information of our patient cohort is summarized in Table 1 and additional details are given in Supplementary Table 1.

### Genomic DNA extraction

Blood DNA matching to the patient cohort was extracted using QIAampBlood Mini Kit (Qiagen, CA, USA) while tumor cryosections collected in microcentrifuge tubes were extracted using the QIAamp AllPrep DNA/RNA Mini (samples from TSH) or QIAamp DNA Mini Kit (samples from KLH, PH, SGH, QESH) according to the manufacturer’s protocols. The extracted DNA samples from both blood and tumor tissues were quantified using the PicoGreen dsDNA Quantitation Reagent (samples from TSH) or the Qubit dsDNA High Sensitivity Assay Kit (samples from KLH, PH, SGH, and QESH).

### Whole exome sequencing

Whole exome sequencing was carried out using tumor and blood DNAs of the Discovery set. Firstly, ~3 μg of DNA was sheared using Covaris focused-ultrasonication (Covaris, MA, USA), followed by end repaired and ligation with paired-end adaptor. The adaptor-ligated libraries were then hybridized with SureSelect Human All Exon 51 Mb probes, followed by exome capture using streptavidin-coated magnetic beads (Agilent Technologies, CA, USA). The captured exomes were then pooled and sequenced using the Illumina Hiseq 2000 platform to generate 91–101 bp paired-end reads to an average coverage of ~100X. The library construction and sequencing were performed by Beijing Genomics Institute (BGI, Shenzhen, China), following their in-house protocols.

### Identification of somatic mutations from exome data

Fastq files of the whole exome sequencing were obtained from BGI for further analysis. Sequencing reads from tumor and matched normal blood DNAs were separately aligned to the human reference genome hg19 using Burrows-Wheeler Aligner^109^. Local realignment was performed using Genome Analysis Toolkit (GATK)^110^, followed by PCR duplicates removal using Picard tool (http://picard.sourceforge.net). Next, variants from both normal and tumor samples were identified using GATK pipeline. Briefly, base qualities were recalibrated, and the GATK UnifiedGenotyper was subsequently employed to call SNVs and Indels. Only well-mapped reads (mapping quality of ≥30 and number of mismatches ≤3 within a 40-bp window) were used as input for the UnifiedGenotyper. Variants that passed additional quality filters (quality by depth of ≥1.5, variant depth of ≥2, total depth ≥10) were retained. To identify somatic mutations (single nucleotide variants [SNVs] and short insertions and deletions [Indels]), variants identified from tumor and matched blood DNA samples were initially compared with dbSNP137, 1000 Genomes Project, Complete Genomic Project (cg69), and National Heart, Lung and Blood Institute (NHLBI; NIH, USA) databases to eliminate any previously reported polymorphisms. Somatic mutations were then identified essentially by subtracting variants in normal DNA samples from the tumor samples and subjected to annotation using ANNOVAR^111^ based on NCBI Refseq database. Only somatic mutations in exons or in splice sites were further analyzed. All potential somatic mutations identified were manually inspected by using the Integrative Genomics Viewer^112^. Amino acid changes were annotated to the longest transcript of the gene, and the impact of somatic SNVs on protein function was predicted using SIFT^113^ and PolyPhen2^114^ (Supplementary Table 3). The putative non-synonymous mutations were analyzed for enriched functional groupings (Gene Ontology classification) using the Database for Annotation Visualization and Integrated Discovery (DAVID)^115^. The list of genes identified as harboring SNVs and Indels were further analyzed and a subset was subsequently selected for external validation using targeted sequencing of the DNA samples from the Prevalence set (described below).

### Haloplex Targeted Sequencing

For targeted sequencing of samples from the Prevalence set comprising of 88 NPC tumors and matched DNAs, a customized Haloplex Target Enrichment Panel (Agilent Technologies) was designed using Agilent SureDesign (https://earray.chem.agilent.com/suredesign). This customized panel covered 99.72% of the targeted region by a total of 19804 amplicons (Total Sequence-able Design Size: 400.037 kbp) and used to capture all exons of the selected 42 candidate genes of interest (37 genes prioritized from exome data and an additional top 5 mutated cancer genes). Briefly, each DNA sample was subjected to eight restriction digest reactions, hybridized with probes against target regions of the selected genes and underwent PCR amplification with Herculase II Fusion Enzyme (Agilent Technologies) to incorporate Illumina paired-end sequencing motifs and index sequences, followed by purification using Agencourt AMPure XP beads (Beckman Coulter, CA, USA). Each captured library were the pooled and subjected to 101-bp paired-end sequencing using the Illumina Hiseq 2000 platform by BGI. Variants from the resulting data were called using the SureCall pipeline (Agilent Technologies). The called variants were then annotated using ANNOVAR^111^ based on NCBI RefSeq database. To identify putative novel variants implicated in NPC pathogenesis, all the polymorphisms reported in dbSNP137, 1000 Genomes Project, Complete Genomic Project (cg69), and NHLBI databases were eliminated. Those coding variants reported in COSMIC database were retained. Only variants that affected the exonic and splicing regions were further analyzed.

### Validation of mutations by Sanger sequencing

From our Discovery set a total of 30 candidate mutations, were selected for validation by Sanger sequencing (Supplementary Table 4). For the Prevalence set, recurrent variants and top 5 most frequently mutated genes were selected for Sanger validation. Somatic or germline status was also determined by Sanger sequencing, where matched blood DNA was available (Supplementary Table 9). Primers specific to the regions of interest harboring the mutations were designed using Primer 3 software (http://www.bioinformatics.nl/primer3plus) and these sequences are listed in Supplementary Table 5. Due to sample limitation, DNA samples from TSH were whole genome amplified using Repli-G Mini kit (Qiagen, GmbH, Germany) prior to use for further PCR amplification for Sanger sequencing. PCR amplification was conducted using Platinum Supermix (Invitrogen, CA, USA) for samples from TSH, or GoTaq Green Master Mix (Promega, Madison USA) for samples from KLH, PH, SGH and QESH. PCR cycling parameters included one cycle at 95 °C for 5 min, 40 cycles at 95 °C for 30 s, 55 °C to 60 °C for 30 s and 72 °C for 1 min, and one cycle at 72 °C for 10 min. Sequencing was performed with ABI BigDye Terminator v3.1 (Life Technologies, CA, USA). The sequence chromatograms were visually inspected with Mutation Surveyor v4.0.4 (Softgenetics, State College, USA), Chromas Lite 2.1.1 or Bioedit software.

## Additional Information

**How to cite this article**: Chow, Y. P. *et al*. Exome Sequencing Identifies Potentially Druggable Mutations in Nasopharyngeal Carcinoma. *Sci. Rep.* **7**, 42980; doi: 10.1038/srep42980 (2017).

**Publisher's note:** Springer Nature remains neutral with regard to jurisdictional claims in published maps and institutional affiliations.

## Supplementary Material

Supplementary Figure 1

Supplementary Tables

## Figures and Tables

**Table 1:** Clinicopathological details of the NPC sample cohort used.

Characteristics	Patients	Percentage
Ethnicity		
Chinese	69	70%
Malay	5	5%
Indigenous	15	15%
Others	9	9%
Gender		
Male	77	79%
Female	21	21%
Age at diagnosis		
Median	51	
≤35	7	7%
36–40	8	8%
41–50	31	32%
51–60	34	35%
>60	17	17%
NA	1	1%
WHO type		
I	1	1%
II	16	16%
III	81	83%
**Clinical stage**		
I	1	1%
II	17	17%
III	48	49%
IVA and IVB	23	23%
IVC (metastasis)	9	9%
