# Supplementary material for: Exome Sequencing Identifies Potentially Druggable Mutations in Nasopharyngeal Carcinoma
Source: Sci Rep. 2017 Mar 3;7:42980. doi: 10.1038/srep42980 (PMC5335658; doi:10.1038/srep42980)
Supplement: Supplementary Figure 1 [file srep42980-s1.pdf]

Title: Exome Sequencing Identifies Potentially Druggable Mutations in Nasopharyngeal Carcinoma

Authors: Yock Ping Chow[1*], Lu Ping Tan[2*], San Jiun Chai[1], Norazlin Abdul Aziz[2], Siew Woh Choo[3], Paul Vey Hong Lim[4], Rajadurai Pathmanathan[5], Noor Kaslina Mohd Kornain[6], Chee Lun Lum[7], Kin Choo Pua[8], Yoke Yeow Yap[9], Tee Yong Tan[10], Soo Hwang Teo[1], Alan Soo-Beng Khoo[2] and Vyomesh Patel[1**]

Supplemental Figure legends:

Supplemental Figure 1. Correlation between NPC tumor stages.and the frequencies of mutations detected in the 4 enriched druggable pathways.

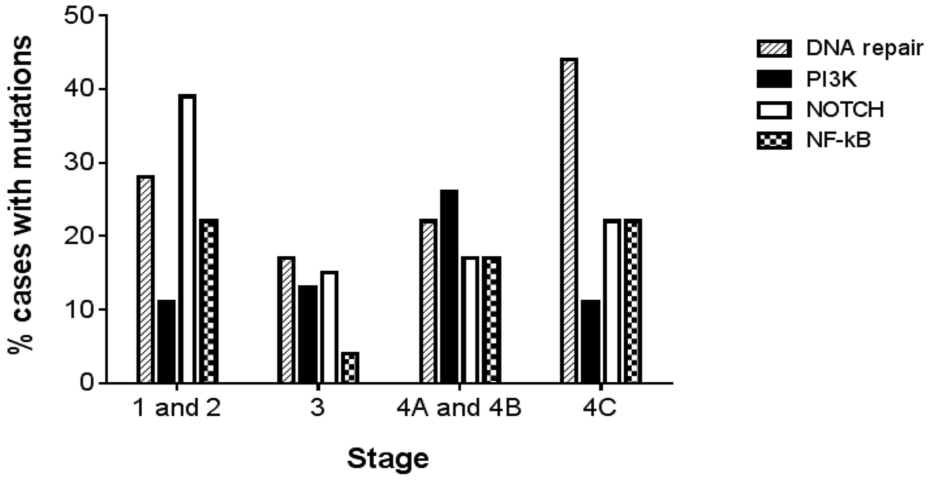

Stage	Number of Cases (Percentage)				
	Total (n=98)	DNA repair	PI3K	NOTCH	NF-κß
1 and 2	18	5/18 (28%)	2/18 (11%)	7/18 (39%)	4/18 (22%)
3	48	8/48 (17%)	6/48 (13%)	7/48 (15%)	2/48 (4%)
4A and 4B	23	5/23 (22%)	6/23 (26%)	4/23 (17%)	4/23 (17%)
4C	9	4/9 (44%)	1/9 (11%)	2/9 (22%)	2/9 (22%)
